# Peritonitis due to colon mantle cell lymphoma

**DOI:** 10.1093/jscr/rjab152

**Published:** 2021-04-24

**Authors:** Rafael Villavicencio, Claudia P Ávalos

**Affiliations:** Department of Surgery, Hospital III Suarez Angamos Essalud, Lima, Peru; Department of Surgery, Hospital III Suarez Angamos Essalud, Lima, Peru

## Abstract

Mantle cell lymphoma is a rare and aggressive type of B-cell non-Hodking lymphoma. It can compromise the gastrointestinal tract, sometimes developing an entity known as multiple lymphomatous polyposis. It develops more frequently in males in the sixth decade of life, presenting heterogeneous clinical patterns. The diagnostic is endoscopic with histological confirmation. Currently, the treatment is chemotherapy, reserving surgical exploration of the abdominal cavity for complicated cases with acute abdomen. We present the case of a 51-year-old woman who underwent emergency surgery to treat peritonitis due perforation of a multiple lymphomatoid polyposis, an unreported atypical complication. It is concluded that although it is an extremely rare entity, it is important to include it in the differential diagnosis of complicated multiple colon polyposis.

## INTRODUCTION

The mantle cell lymphoma (MCL) a rare type of non-Hodgkin lymphoma, characterized by overexpression of protein cyclin D1 on chromosome 11 [1]. It presents as advanced disease with clinical variable, mainly affecting lymph nodes, up to 80% show extranodal involvement; of this, 20% affect gastrointestinal tract presenting a mass or numerous smalls polyps called multiple lymphomatous polyposis (MLP) [2]. Since the name MLP was given to this disease in 1961 [3], very few cases have been reported. We present a patient treated with surgery in a local hospital for extensive colorectal MLP complicated with peritonitis.

## CASE PRESENTATION

**Figure 1:**
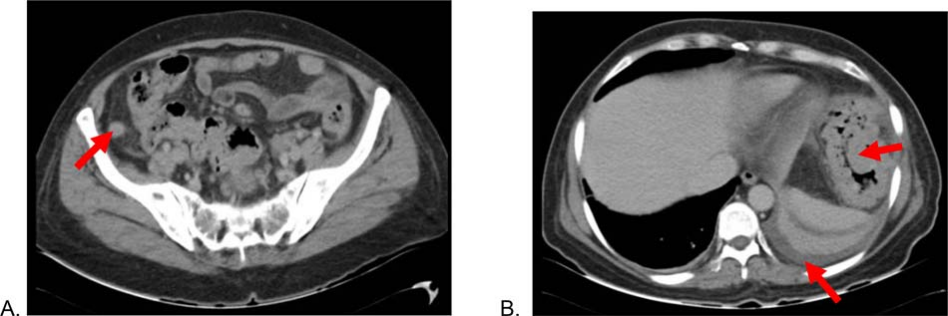
Abdominal tomography: (**A**) Thickened cecal appendix and (**B**) Perisplenic free fluid, splenic flexure of the colon with exophytic tumor.

**Figure 2:**
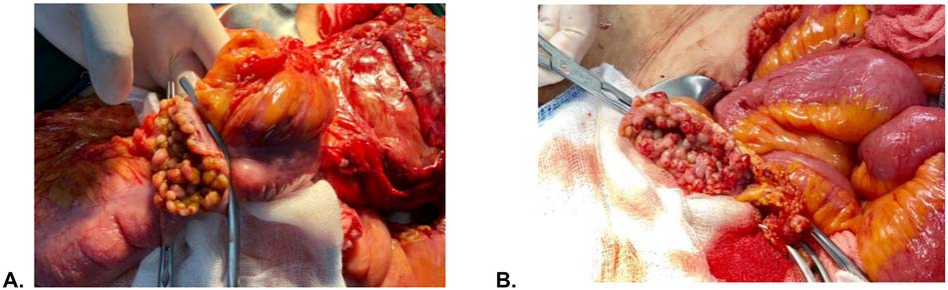
Intraoperative findings. (**A**) Colonic mucosa and (**B**) rectal stump mucosa.

A 51-year-old female patient with a history of uterine polyp treated with curettage 2 years ago and use of omeprazole for gastritis without endoscopic confirmation. A total of 15 days before admission presents mild pain localized in superior hemiabdomen associated with nausea, liquid stools and fever; abdominal pain became diffuse and intensified 2 days before admission, so she goes to emergency room. Physical examination revealed tachycardia, decreased vesicular murmur at base of left hemithorax, slightly distended abdomen, very painful to palpation, positives Mc Burney and rebound signs. Lab test showed significant leukocytosis with left deviation, mild anemia and hypoalbuminemia. The tomography shows thickened cecal appendix, enlarged ileocolic nodes, free fluid around the spleen and splenic flexure of the colon with exophytic tumor (Fig. 1), then diagnostic laparoscopy was decided. A total of 1000 cc of purulent fluid was found throughout the abdominal cavity, thickened cecal appendix but not inflammatory signs, splenic flexure of colon with an inflammatory-appearing tumor with abundant fibrin and multiple paracolic nodes, so laparotomy was decided. During the intestinal section evidenced colorectal mucosa with multiple polyps (Fig. 2), we performed total colectomy and terminal ileostomy for probable familial adenomatous polyposis, patient progresses favorably, is discharged from surgery and referred to medical oncology for management.

**Figure 3:**
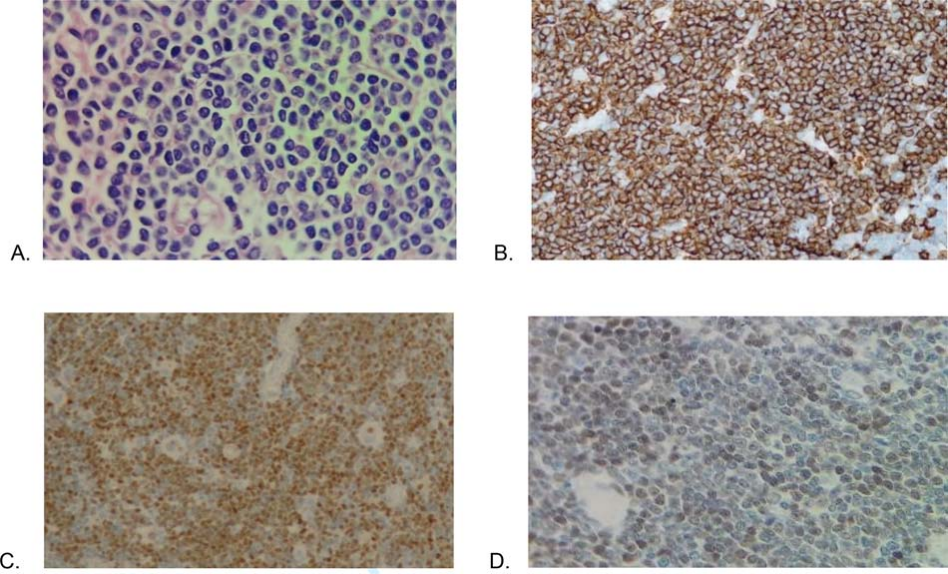
Histopathology (**A**) Proliferation of atypical lymphoid cells in colonic mucosa and submucosa (hematoxylin eosin—×100), (**B**) CD20 positive, (**C**) cyclin D1 positive and (**D**) SOX11 positive.

Microscopy shows MCL with a histological pattern of lymphomatoid polyposis, which involves mucosa of colon and ileum, extending to muscularis propria and to subserous layer focally. Immunohistochemistry showed positive for CD20, CD3 (in T cells), CD79a, CD43, BCL2, CYCLIN D1, SOX11, and proliferative index 50% at Ki67 (Fig. 3), IgG VCA antibodies and antibody for Epstein Barr Virus were reactive at 662.12 and 16.7 U/ml, respectively. Tumor markers were within normal parameters. With the diagnosis of Mantle cell non-Hodking lymphoma, the primary of gastrointestinal tract, clinical stage IIE, MIPI 3 scale, chemotherapy combined with R-CHOP (Rituximab, cyclophosphamide, vincristine, doxorubicin and prednisone) was started after six courses presented progression in rectal mucosa biopsy, then start 3 cycles of RICE scheme (rituximab, ifosfamide, carboplatin and etoposide), undergoing autologous bone marrow transplantation twice, after patient shows positron emission tomography (PET) scan without evidence of metabolic activity compatible with recurrence and new rectal biopsy shows regenerative erosion of epithelium without evidence of lymphoproliferative activity.

## DISCUSSION

Colon lymphomas represent 0.4% of colon neoplasms [4]. The cell lymphoma mantle is a rare and aggressive subtype of B-cell non-Hodgkin lymphoma caused by overexpression of the cyclin D1 protein on chromosome 11 due to t translocation (11,14) (q13; q23) [1]. Classically affect lymph nodes, up to 80% of patients show extra-nodal involvement, depending on the molecular pathways involved in development [5]. It affects three times more men with an average age of 60 years. Mostly asymptomatic, but may have abdominal pain, diarrhea, weight loss, fatigue and night sweats; it can even lead to protein-losing enteropathy, chylous ascites and intestinal malabsorption [6]. It is very rare that it causes acute abdomen; when it does, it is due to ileocecal intussusception, hemorrhage or perforation [6, 7]. Enteroscopy and colonoscopy are important in the diagnosis; it provides polyps localization and possibility of obtaining biopsies. MCL has a median survival of 4 years after diagnosis, with a long-term survival rate of <15% [8]. In gastrointestinal tract, it can present as a mass, especially in ileocecal region, but any other area can be involved from stomach to rectum. It also develops with presence of multiple polypoid lesions of variable diameter, following a segmental or global distribution, receiving the name of multiple lymphomatoid polyposis; this MLP affects the stomach in 64%, small intestine and colon in up to 90% and rectum in 52% of cases [7]. The treatment is chemotherapy, which achieves complete response rates of 30–50% [2]. In the case of complications, emergency surgery with subsequent chemotherapy is indicated [9].

This case report is important for the management of acute abdomen due to complicated polyposis. Knowledge of differential diagnoses is extremely important because not all MLPs are the result of MCL [10], must be taken into account other options such as familial adenomatous polyposis, Peutz–Jeghers syndrome, colorectal carcinoma, gastrointestinal lipomatosis, atypical adenoma and lymphoid nodular hyperplasia with hypogammaglobulinemia [5]. So, although most of polyposis mentioned require extensive surgeries, the MCL does not benefit from a wide resection; however, its infrequency and the lack of an adequate intraoperative study would not allow it to be conservative.

## CONFLICT OF INTEREST STATEMENT

The authors declare no conflicts of interest.

## FUNDING

Own financing.

## CONSENT FOR PUBLICATION

Written informed consent was obtained from the patient for publication of this case report.
